# Ethanol locks for the prevention of catheter-related infection in patients with central venous catheter: A systematic review and meta-analysis of randomized controlled trials

**DOI:** 10.1371/journal.pone.0222408

**Published:** 2019-09-12

**Authors:** Jun Zhang, Bo Wang, Jinxia Wang, Qin Yang

**Affiliations:** 1 School of Nursing, Gansu University of Chinese Medicine, Lanzhou, Gansu, China; 2 Department of Special Surgery, the First Hospital of Lanzhou University, Lanzhou, Gansu, China; University Magna Graecia of Catanzaro, ITALY

## Abstract

**Background:**

The widespread use of central venous catheters (CVCs) has exposed patients to a high risk of catheter-related infection (CRI), which is linked to substantial morbidity and mortality. Several strategies for preventing CRI, including ethanol lock prophylaxis, have been explored. This study aimed to provide a comprehensive summary of randomized controlled trials (RCTs) assessing the efficacy and safety of ethanol locks for preventing CRI in patients with CVC.

**Methods:**

We searched six electronic databases, earlier relevant meta-analyses and the reference lists of the included studies for RCTs that assessed the effects of ethanol locks on CRI in patients with CVC versus a control group. Two authors independently assessed the methodological quality of the included studies using the Cochrane Risk of Bias tool and extracted relevant information according to a predesigned extraction form. Data were analyzed using the Cochrane Collaboration’s RevMan 5.3.

**Results:**

Nine studies involving 2451 patients were included. Although limited in power, the results of the meta-analysis indicated a positive effect of ethanol lock prophylaxis on reducing catheter-related bloodstream infection (CRBSI) compared to heparin alone [OR = 0.53, 95% CI 0.34, 0.82, *P* = 0.004]. The effects on other outcomes, such as exit site infection, catheter dysfunction, catheter removal, thrombosis and mortality, were not statistically significant (*P* > 0.05). Moreover, although the effect of ethanol on CRBSI was in the expected direction compared to 0.9% NaCl locks, this effect was not statistically significant (*P* > 0.05).

**Conclusions:**

The present data indicate that ethanol lock prophylaxis is a potential candidate for the prevention of CRBSI in patients with CVC. However, more attention should be paid to the uniform ethanol lock procedure and toxic effects after long-term ethanol lock exposure.

## Introduction

Central venous catheter (CVC) is an indispensable lifesaving intervention for critically ill adults and pediatric patients requiring treatments such as chemotherapy, parenteral alimentation, hemodialysis and treatment for hematological malignancies. The widespread use of CVC exposes patients to a high risk of catheter-related infection (CRI), which includes catheter-related bloodstream infection (CRBSI) and exit site infection. Despite improved international guidelines on CVC placement and catheter care, CRI remains a major complication. As reported in the literature, 10% to 20% of tunneled catheters become infected in patients receiving chemotherapy for hematological malignancy[1,2]. Among all parenteral nutrition patients, the incidence of CRBSI ranges from 0.6 per 1000 catheter days to 5.36 per 1000 catheter days[3]. Similarly, it has been estimated that the relative risk (RR) for infection in hemodialysis catheters compared with native arteriovenous fistulae is 15.5 and 25.5[4].

CRI can inevitably cause substantial morbidity and mortality, which leads to extended hospital admissions and increased health care costs[5,6]. Thus, preventing CRI is a real clinical challenge, and several strategies have been adopted to reduce its incidence. In recent years, several studies have employed specific locking solutions instead of the standard heparin locks. A promising approach is the use of antibiotic lock solutions for the catheter to prevent intraluminal colonization and the development of biofilm. Decreased CRI rates have been reported by many clinical studies and confirmed in recent meta-analyses[7–9]. However, concerns have been raised regarding antibiotic lock solutions, given the particular risk of causing microbial resistance, although no substantial evidence has been published to date. Therefore, the preventive use of antibiotics should be avoided if alternative options exist.

For this purpose, ethanol is a potential candidate for the prevention of CRI. It is an easily available antiseptic with a broad antimicrobial spectrum, no known acquired resistance and minimal adverse effects. In the past few years, an increasing number of clinical studies investigating this approach have attested to the benefits of ethanol locks for CRI prevention[10–12]. Data from the relevant literature showed that ethanol locks were beneficial for reducing the occurrence of CRI. In addition, a few systematic reviews/meta-analyses regarding this prophylaxis have been published[13,14]. Similarly, these analyses have concluded that ethanol lock solutions reduce the risk of CRI for patients with CVCs. However, these studies are limited by some methodological problems, which can result in bias risk. For example, Zhao et al concluded that ethanol lock is effective in reducing the incidence of CRBSI in hemodialysis patients[13]. However, in their meta-analysis, the majority of participants in the included studies were not hemodialysis patients. Zhang et al[14] pooled all of the data directly in their meta-analysis and did not distinguish the types of control used in each domain. Furthermore, a decision regarding the use of ethanol locks should be based on the sustainability of catheters and potential adverse events, which was not reported fully in either of these two meta-analyses[13,14]. In this case, we believe that it is still necessary to carry out studies in this field. Thus, to further clarify the efficacy and safety of ethanol locks for the prevention of CRI, we conducted this systematic review and meta-analysis based on these existing RCTs.

## Materials and methods

### Protocol and registration

This protocol has been registered in PROSPERO (http://www.crd.york.ac.uk/PROSPERO/) under registration number CRD42015027833.

### Eligibility criteria

#### Type of study

Any relevant RCTs were included. For studies with the same or overlapping data by the same authors, the most appropriate studies with the largest number of cases or most recent publication dates were selected.

#### Participants

The participants were adults and children with a tunneled or nontunneled CVC as vascular access, regardless of the type of disease.

#### Type of interventions

Ethanol lock solutions were used in the intervention group. Solutions were allowed to dwell rather than simply being flushed through the catheter. A control condition (e.g., heparin locks) was used in the control group.

#### Outcomes of interest

The primary outcome was CRBSI (as defined by the study author). The secondary outcomes were exit site infection (defined as the development of purulent exudates or redness around the site not resulting from residual stitches), catheter dysfunction (defined as catheter blockage or persistent inadequate flow rate), removal of the catheter (defined as removal of the catheter for any reason before the end of prophylactic treatment), catheter thrombosis (defined as thrombosis or the need for thrombolytic therapy or removal of the catheter because of thrombosis), and all-cause mortality and adverse events as reported by the study author. Incidence was presented as the number of episodes per catheter-day.

### Information sources and search

We systematically performed an electronic search of PubMed, the Cochrane Library, EMBASE (via the Embase.com platform), Sciences Citation Index (via the Web of Knowledge platform), the Chinese Biomedical Literature Database and the Chinese Digital Journals Full-text Database from their inception to March 2018 with no language restrictions. In addition, we searched unpublished theses and dissertations via the Conference Proceedings Citation Index, China Proceeding of Conference Full-text Database, China Doctoral Dissertation Full-text Database and China Master’s Theses Full-text Database. Relevant systematic reviews and meta-analyses from these databases were identified, and their bibliographies were scrutinized for further relevant trials, as were those of the RCTs included in the review. The search method included relevant text words and medical subject headings related to ethanol, infection, CVC and RCT. The exact search strategy used in the PubMed database is provided as an example in S1 text.

### Study selection

Literature search results were imported into ENDNOTE X7 literature management software. Two authors independently reviewed the title, abstract or descriptors of the identified studies and excluded studies that clearly did not meet the inclusion criteria. After excluding duplicate and apparently irrelevant studies, the full text of the remaining studies was reviewed to assess eligibility for inclusion. Any disagreements were resolved by discussion or by seeking an independent third opinion. Excluded trials and the reason for their exclusion were listed and examined by a third reviewer.

### Data collection process and data items

Two authors independently extracted the data from each study using a standardized data extraction checklist, which included study characteristics (e.g., first author’s name, publication year, journal, country where the study was conducted), characteristics of the study subjects (e.g., type of disease, number of participants, age, sex distribution), characteristics of the catheter (e.g., type of catheters, number of catheters, site of catheter insertion), interventions details (e.g., type of lock solutions, patient involvement, number of catheter days), outcome variables (e.g., number of episodes) and any additional prophylactic measures used that may have affected outcomes (e.g., catheter care). Outcomes were extracted preferentially by intention to treat (ITT) at the end of follow-up. Quantitative data were extracted to calculate effect sizes. Data on effect size that could not be obtained directly were recalculated when possible. Any discrepancy was resolved by consensus.

### Risk of bias within individual studies

Two authors independently evaluated the methodological quality of the included studies for major potential sources of bias using the Cochrane Collaboration’s risk of bias tool[15], which includes the method of random sequence generation, allocation concealment, blinding of participants and personnel, blinding of outcome assessment, incomplete outcome data, selective reporting, and other sources of bias. We evaluated the methodological quality of each study by rating each criterion as low, high or unclear risk of bias. Any disagreements were resolved through discussion with another reviewer if needed.

### Statistical analysis

Review Manager Software (RevMan 5.3) was used for meta-analysis. We assessed clinical and methodological heterogeneity by carefully examining the characteristics and design of the included trials. The I^2^ statistic was used to assess heterogeneity among the studies, and values over 50% were considered to represent high heterogeneity. A fixed-effects model was used to pool the results unless significant heterogeneity was observed (I^2^> 50%), which required a random-effects model. All of the variables in the included studies were dichotomous data, so we used an odds ratio (OR) with a 95% confidence interval (CI) to analyze the effect size of the studies. Subgroup analysis was planned to assess the potential sources of heterogeneity (e.g., type of disease, type of catheter). We described the results of the original studies if the data could not be extracted to calculate the total effect.

## Results

### Study selection

**Fig 1:**
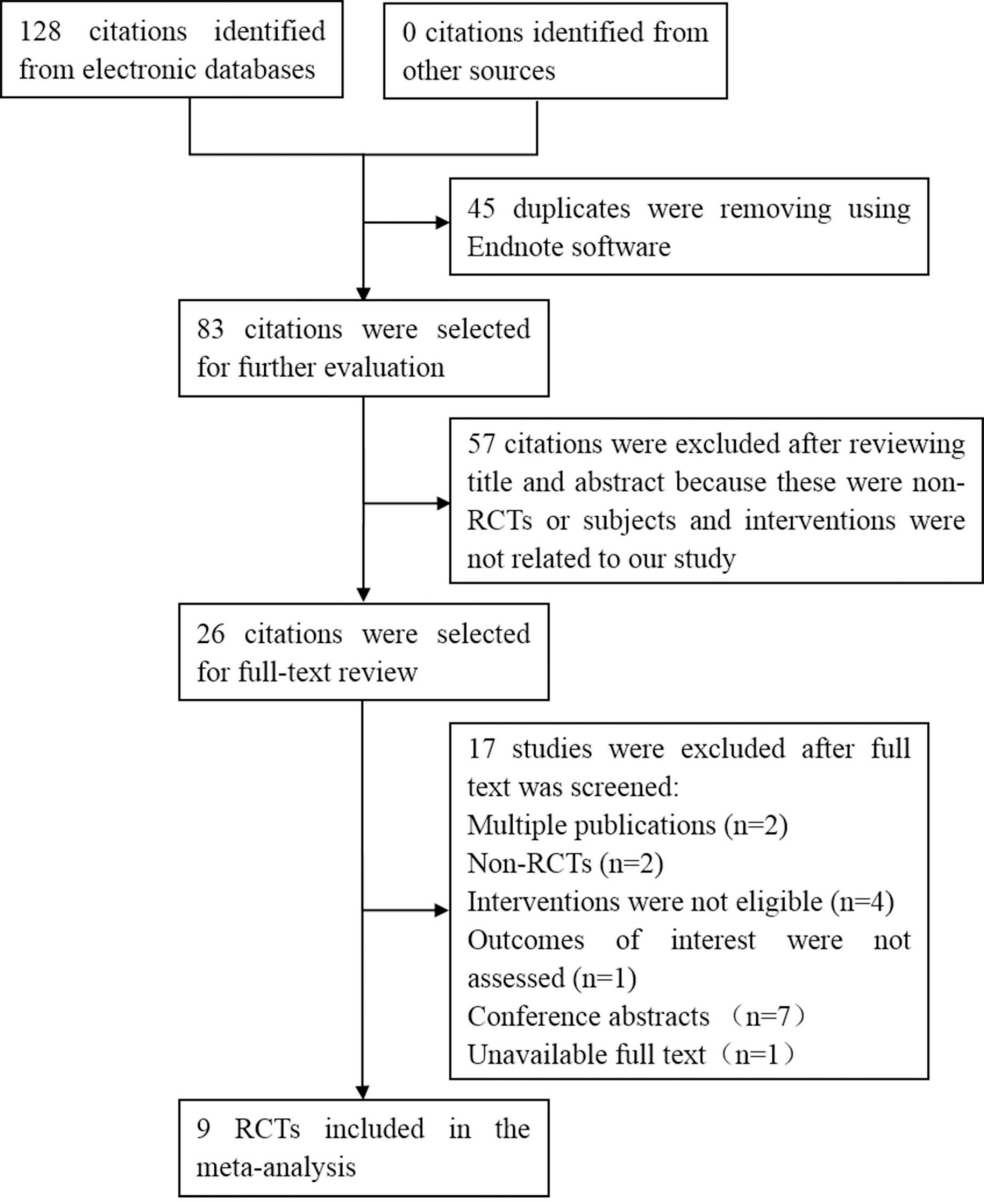
Flow diagram of included and excluded studies.

Fig 1 presents a flow diagram illustrating the study selection process. The electronic searches identified 128 studies, of which 45 duplicates were excluded by Endnote software and 57 articles were clearly not relevant after the first screening. The full text of twenty-six studies was retrieved in-depth consideration, and 17 studies were excluded for the following reasons: multiple publications (n = 2), non-RCTs (n = 2), ineligible interventions (n = 4), nonassessment of outcomes of interest (n = 1), unavailable conference papers (n = 7) and unavailable full text (to date, we have been unable to obtain more information about this study although we have contacted the authors). Finally, 9 studies[16–24] were included in our systematic review. References cited in published original and review papers were examined, and no further studies were found.

### Characteristics of the included studies

The characteristics of the studies included in the systematic review are presented in Table 1. Of these included studies, two were performed in Australia[18,21], two in China[19,20], two in the Netherlands[17,22], one in the USA[24], one in France[23] and one in New Zealand[16]. The combined sample size across the 9 included studies was 2451 participants with a total of 3235 catheters[16–24]. The patient samples in the included studies were variable. Three included hematology patients[16,17,21], three included hemodialysis patients[18–20], one focused on pediatric oncology patients[22], one comprised parenteral nutrition patients[24], and one comprised renal-replacement therapy and plasma exchange patients[23]. All studies randomized patients to ethanol lock solutions, which were compared to either heparin or 0.9% NaCl. The concentration of the lock solutions used in individual studies is presented in S1 Table. In the studies we reviewed, various ethanol lock procedures were used (Table 2). Lock volume ranged from 1.5 to 3.3 ml. Lock dwell time varied among the studies, ranging from 2 minutes to 48 hours. In these studies, the most common catheters used were tunneled catheters, which were employed in 6 trials[16–18,20–22]. The location of the dialysis catheter was reported in 8 trials[16–23], with the majority being inserted in the internal jugular and subclavian veins (S1 Table). The criteria used for CRBSI diagnosis were detailed in 8 trials[16–18,20–24]. In these studies, diagnostic criteria provided by different organizations were used. However, all criteria used for CRBSI diagnosis are based on laboratory-confirmed results. Additionally, catheter care is of crucial importance to patients using intravascular catheters. Only four studies[16,18,20,23] described catheter care procedures. Cleaning the catheter site and changing the dressing at each use appeared to be the most common type of catheter care.

**Table 1:** Characteristic of the included studies.

First author, Year	Country	Disease	No. of patients (Exp/control)	No. of catheters (Exp/control)	Tunneled or nontunneled catheter	No. of catheters days (Exp/control)	Intervention	
							Exp	Control
Sanders 2008[16]	New Zealand	hematology	34/30	34/30	tunneled	5000/3537	70% ethanol	heparin
Slobbe 2010[17]	Netherlands	hematology	376	226/222	tunneled	14262/13483	70% ethanol	0.9% NaCl
Broom 2012[18]	Australia	hemodialysis	25/24	25/24	tunneled	3614/1834	70% ethanol	heparin
Yang 2013[19]	China	hemodialysis	20/20	20/20	not reported	not reported	70% ethanol	heparin
Sun 2014[20]	China	hemodialysis	16/16	16/16	tunneled	4449/4363	70% ethanol	heparin
Worth 2014[21]	Australia	hematology	42/43	42/43	tunneled	2216/2657	70% ethanol	heparin
Schoot 2015[22]	Netherlands	pediatric oncology	153/154	153/154	tunneled	20916/19915	70% ethanol	heparin
Souweine 2015[23]	France	renal-replacement therapy or plasma exchange	730/730	1106/1066	nontunneled	6541/6496	60% wt/wt ethanol	0.9% NaCl
Salonen 2017[24]	USA	parenteral nutrition	18/20	18/20	not reported	2597/ 3125	70% ethanol	heparin

Exp, experimental group; Control, control group

**Table 2:** Intervention protocols of the included studies.

First Author, Year	Ethanol lock procedure
Sanders 2008[16]	Three milliliters of 70% ethanol was injected into each lumen of the catheter daily and left for 2 hours before being entirely removed and replaced with heparinized saline.
Slobbe 2010[17]	During hospitalization, every lumen of the CVC was locked with 3 ml 70% ethanol for 15 minutes per day, following which the solution was flushed through with 10 ml 0.9% NaCl. During outpatient settings, ethanol locks were administered once weekly before the replacement of the regular heparin solution.
Broom 2012[18]	Participants received weekly catheter instillation of 3 ml 70% ethanol for 48 hours together with standard heparin locks following the remaining 2 hemodialysis sessions each week.
Yang 2013[19]	Seventy percent ethanol was instilled into each lumen of the CVC weekly.
Sun 2014[20]	After flushing CVC lumens with 20 ml 0.9% NaCl at the end of a hemodialysis session, 3.3 ml 70% ethanol was instilled into each catheter lumen and left in situ until the next dialysis session, when it was aspirated.
Worth 2014[21]	After flushing CVC lumens with 10 ml 0.9% NaCl, 2 ml 70% ethanol was instilled into each CVC lumen daily for inpatients and left in situ for 2 hours. A 5- to 10-ml aliquot was then aspirated from each lumen before locking under positive pressure with 10 mL 0.9% NaCl. Self-caring outpatients were instructed to administer the ethanol lock three times weekly, with 2 hours dwell time.
Schoot 2015[22]	Based on the size of the CVC, 1.5 or 3.0 ml of 70% ethanol was injected into each lumen of the catheter. After two hours, the ethanol lock solution was flushed with 0.9% NaCl, and the CVC was closed with heparin. Locks were administered at least once every six weeks, but with a maximum lock frequency of once weekly.
Souweine 2015[23]	Two milliliters of ethanol was injected into each catheter lumen and left for 2 minutes before being entirely removed. Each lumen was then flushed with 20 ml of 0.9% NaCl and locked during the interrenal-replacement/plasma exchange periods with 0.9% NaCl containing 100 U/ml of unfractionated heparin.
Salonen 2017[24]	Patients flushed their catheters with 10 ml 0.9% NaCl after completion of their parenteral nutrition and then locked the catheter with 3 ml 70% ethanol. Prior to administration of the next bag of parenteral nutrition, they again flushed their catheters with 10 mL 0.9% NaCl.

### Methodological quality of the included studies

Table 3 shows the quality assessment of the studies in this systematic review. All 9 included studies[16–24] were randomized, but only six[16–18,20,22,24] specified the method of randomization. Four studies[16–18,22] depicted the allocation concealment, while the other studies did not reference whether any allocation concealment process was used. Three trials[17,22,24] blinded patients, personnel and outcome assessors. The patients and personnel providers alone were blinded in 2 trials[16,23]. Five studies reported withdrawals[16,17,20,22,23], and the remainder had no apparent dropouts. Intention to treat (ITT) was performed for all patients.

**Table 3:** Methodological quality of the included studies.

First Author, Year	Random sequence generation	Allocation concealment	Blinding of participants and personnel	Blinding of outcome assessment	Incomplete outcome data	Selective reporting	Other source of bias
Sanders 2008[16]	Computer-generated	Pharmacy	Low risk	High risk	Low risk	Low risk	Unclear
Slobbe 2010[17]	Computer-generated	Pharmacy	Low risk	Low risk	Low risk	Low risk	Unclear
Broom 2012[18]	Computer-generated	Central distribution	High risk	High risk	Low risk	Low risk	Unclear
Yang 2013[19]	Unclear	Unclear	High risk	High risk	Low risk	Unclear	Unclear
Sun 2014[20]	Computer-generated	Unclear	High risk	High risk	Low risk	Low risk	Unclear
Worth 2014[21]	Unclear	Unclear	High risk	High risk	Low risk	Unclear	Unclear
Schoot 2015[22]	Computer-generated	Central distribution	Low risk	Low risk	Low risk	Low risk	Unclear
Souweine 2015[23]	Unclear	Unclear	Low risk	High risk	Low risk	Low risk	Unclear
Salonen 2017[24]	Computer-generated	Unclear	Low risk	Low risk	Low risk	Low risk	Unclear

### Meta-analysis

#### Ethanol vs heparin

**Fig 2:**
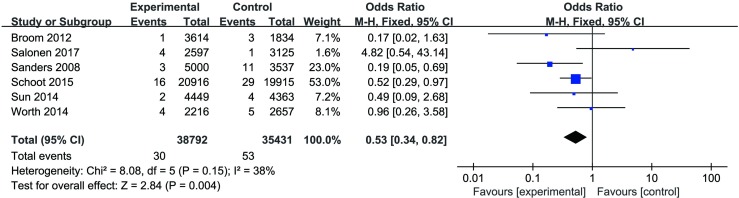
CRBSI per 1000 catheter days for trials that compared ethanol locks with heparin.

CRBSI. A total of 7 trials[16,18–22,24] (615 participants, 615 catheters) reported this outcome, but we were unable to include the study conducted by Yang[19] in our analysis because of missing data for catheter days; consequently, only 6 studies[16,19–22,24] were included in the data pooling. The fixed-effects model was used because heterogeneity was not evident among these studies (*P* = 0.15, I^2^ = 38%). A statistically significant difference was observed (OR = 0.53, 95% CI 0.34, 0.82, *P* = 0.004), which indicated that ethanol lock solutions were more effective in reducing CRBSI when compared to heparin alone (Fig 2).

Exit site infection. Four studies[16,18,20,21] involving 230 participants (230 catheters) were included in this comparison. The fixed-effects model was applied because no significant heterogeneity was found (*P* = 0.89, I^2^ = 0%). The pooled analysis of these studies indicated that there was no significant difference between the two groups (OR = 0.88, 95% CI 0.28, 2.81, *P* = 0.83) (S1 Fig).

Catheter dysfunction. Four studies[18,19,20,22] reported this outcome, but we were unable to include the study conducted by Yang[19] in our analysis because of missing data for catheter days; therefore, only 3 studies[18,20,22] (388 participants, 388 catheters) were included in the data pooling. The fixed-effects model was used because heterogeneity was low among these studies (*P* = 0.34, I^2^ = 8%). No significant difference was observed between the groups (OR = 0.73, 95% CI 0.33, 1.62, *P* = 0.43) (S2 Fig).

Catheter removal. Two studies[16,22] involving 371 patients with 371 catheters were entered into the analysis of the effects of intervention on catheter removal. The fixed-effects model was used because the heterogeneity test showed an I^2^ of 0% among studies. No significant difference was observed between the groups (OR = 0.70, 95% CI 0.38, 1.28, *P* = 0.25) (S3 Fig).

Thrombosis. Four studies[16,18,21,22] involving 505 patients with 505 catheters contributed to the analysis of thrombosis. There was no heterogeneity among the trials (*P* = 0.48, I^2^ = 0%); therefore, we used the fixed-effects model for the pooled estimate. The pooled data showed that the occurrence of thrombosis was not significantly different between the groups (OR 0.96, 95% CI 0.21, 4.42, *P* = 0.96) (S4 Fig).

Mortality. Four studies[16,20,22,24] involving 441 patients reported mortality. A fixed-effects model was applied because no statistical heterogeneity was indicated (*P* = 0.92, I^2^ = 0%). The results of the meta-analysis showed that there was no significant difference between ethanol and heparin (OR 0.58, 95% CI 0.16, 2.02, *P* = 0.39) (S5 Fig).

#### Ethanol VS 0.9% NaCl

CRBSI. Two studies[17,23] involving 1836 participants with 2620 catheters reported this outcome. There was no heterogeneity among the trials (*P* = 0.42, I^2^ = 0%); therefore, we used the fixed-effects model for the pooled estimate. The pooled analysis of these studies indicated that there was no significant difference between the two groups (OR 0.74, 95% CI 0.43, 1.28, *P* = 0.28) (S6 Fig).

Mortality. Two studies[17,23] involving 1836 participants were included in this comparison. A fixed-effects model was applied because no statistical heterogeneity was indicated (*P* = 0.52, I^2^ = 0%). No significant difference was observed between ethanol and 0.9% NaCl (OR = 0.95, 95% CI, 0.78, 1.17, *P* = 0.64) (S7 Fig).

### Sensitivity analyses

**Fig 3:**
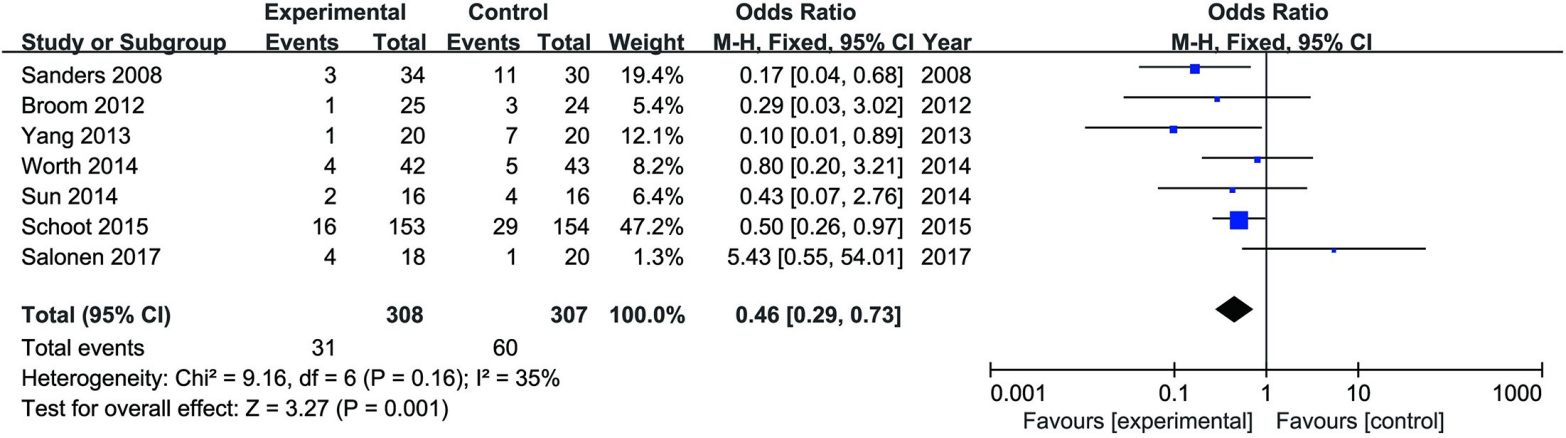
CRBSI per patient for trials that compared ethanol locks with heparin.

To assess the robustness of the conclusions, a sensitivity analysis was conducted to reanalyze the effect of ethanol locks on the primary outcome when expressed as episodes per patient. A total of 7 trials[16,18–22,24] including 615 patients were included in the analysis. The pooled results showed a statistically significant difference between the two groups in favor of ethanol (OR = 0.46, 95% CI 0.29, 0.73, *P* = 0.001) (Fig 3). In addition, in the trial by Schoot et al[22], the participants were pediatric patients, which differed from the other six studies[16,18–21,24]. Therefore, we excluded this study to perform the sensitivity analysis to assess the robustness of the conclusions to the quality of the data. After the sensitivity analysis, the results were unchanged, and there was still a statistically significant difference in CRBSI between the groups. No significant difference was observed between the two groups in terms of catheter dysfunction (OR = 0.56, 95% CI 0.23, 1.36, *P* = 0.20), thrombosis (OR = 0.63, 95% CI 0.10, 4.03, *P* = 0.62) and mortality (OR = 0.69, 95% CI 0.11, 4.42, *P* = 0.70). We did not recalculate effect sizes for catheter removal in this analysis due to the small number of studies that examined this outcome.

### Adverse events

A summary of the adverse events reported in the included trials is presented in Table 4. There were only two trials[23,24] that claimed no adverse effects related to ethanol. The most commonly reported adverse events among the ethanol therapies were transient reactions, such as facial flushing, nausea/vomiting, dizziness/drowsiness and altered taste, which usually subsided in a few days. Sanders et al[16] reported that one patient in the ethanol group experienced an episode of dyspnea immediately after the first treatment and withdrew from the study. Slobbe et al[17] reported that one patient in the ethanol group had syncope shortly after flushing the first lock solution, but no further adverse effects occurred in this particular patient during subsequent ethanol lock procedures. Additionally, no life-threatening adverse events were observed.

**Table 4:** Adverse events reported in the included trials.

First Author, Year	Adverse events	
	Exp	Control
Sanders 2008[16]	Dyspnea immediately after the first treatment (n = 1)	Unusual taste sensation and anxiety (n = 1)
Slobbe 2010[17]	Facial flushing (n = 39); nausea/vomiting (n = 20); altered taste (n = 31); dizziness/drowsiness (n = 41); syncope shortly after the first treatment (n = 1)	Facial flushing (n = 17); nausea/vomiting (n = 17); altered taste (n = 19); dizziness/drowsiness (n = 10)
Broom 2012[18]	Stinging at the catheter exit site (n = 1); dry lips and thirst (n = 1)	Bleeding (n = 1)
Yang 2013[19]	Dizziness/drowsiness (n = 1)	No adverse events
Sun 2014[20]	Facial flushing (n = 1); bleeding after insertion (n = 1); bad smell (n = 1)	Bleeding (n = 4)
Worth 2014[21]	Chest discomfort (3); nausea (n = 1)	No adverse events
Schoot 2015[22]	Nausea (n = 28); vomiting (n = 12); altered taste (n = 89); dizziness (n = 19); flushing (n = 31); drowsiness (n = 8); pain with injection (n = 8)	Nausea (n = 14); vomiting (n = 9); altered taste (n = 26); dizziness (n = 4); flushing (n = 4); drowsiness (n = 3); pain with injection (n = 4)
Souweine 2015[23]	No adverse events	No adverse events
Salonen 2017[24]	No adverse events	No adverse events

Exp, experimental group; Control, control group

## Discussion

This meta-analytic review provided a quantitative summary of the currently available RCTs assessing the efficacy and safety of ethanol lock solutions for patients with CVC.

### Summary of the results

Based on data from 7 RCTs[16,18–22,24] that compared ethanol lock therapy to the current standard of care with heparin, the combined results showed a statistically significant effect size for CRBSI; however, there were no statistically significant differences between the two groups in other outcomes, such as exit site infection, catheter dysfunction, catheter removal, thrombosis, and mortality. In addition, in this meta-analysis, 2 studies[17,23] assessed the effect of ethanol compared with 0.9% NaCl locks. The pooled data showed a downward trend in the rate of CRBSI; however, this trend did not reach statistical significance. The results of the sensitivity analyses that excluded trials of pediatric patients showed broad agreement with the main analyses. On the whole, our results are in accordance with previously published systematic reviews [13,14,25,26]. In this case, our study adds to these previous efforts by providing an RCT-based confirmation of the efficacy of ethanol lock solutions for decreasing CRBSI.

As described in detail previously, CRI results from the migration of skin organisms along the catheter into the bloodstream or the contamination and colonization of catheter lumens[27]. Prevention strategies are directed at decreasing the growth and adherence of pathogens to the catheter hub and surface [27]. In vitro studies have demonstrated the efficacy of ethanol as a lock solution for the eradication of various pathogens that commonly cause CRBSI and for the prevention of biofilm formation[28,29]. Exit site infections are an additional cause of morbidity in patients with CVCs and may contribute to the pathogenesis of CRBSI [30]. In the studies included in our meta-analysis, a low incidence of exit site infections was observed with no difference between the ethanol lock group and heparin group, as expected.

It is notable that with the accumulation of studies supporting the efficacy of ethanol lock prophylaxis for CRBSI, concern has been raised regarding the particular risk of thrombosis with the ethanol lock solution. Ethanol appears to possess intrinsic anticoagulant activity. In some clinical practices, ethanol has been used as an anticoagulative agent and described as a substitute for heparin to maintain catheter patency[31,32]. In our study, there was no significant difference in the occurrence of thrombosis between the two arms.

Catheter integrity after ethanol lock exposure is another topic of interest in this field. In vitro studies have demonstrated that ethanol has a negligible impact on the mechanical properties of catheters[33,34]. In the current meta-analysis, no statistically significant effects were observed from the pooling data on catheter dysfunction and catheter removal. Whereas Mermel et al interpreted the literature as suggesting that the use of ethanol locks may be associated with catheter damage[35]. One explanation is that their finding on this problem was largely based on retrospective studies and qualitative analysis.

All-cause mortality was not affected by ethanol lock therapy. No significant difference was detected when comparing ethanol with heparin or 0.9% NaCl. Adverse events were described in the meta-analysis. Generally, the incidence of adverse reactions was tolerable across all included studies.

In this meta-analysis, although a lower incidence of CRBSI was observed in preventive ethanol locks when compared to 0.9% NaCl, the difference was nonsignificant. The minimal impacts may be explained by the small number of studies included in this comparison and the low event rates. In addition, for practical reasons, the duration of ethanol locks was short. In Slobbe et al’s study [17], they used a lock time of 15 minutes daily per catheter lumen. Souweine et al’s ethanol lock dwell time was only 2 minutes [23]. In other studies [16,18–22,24], the dwell time was at least 2 hours.

### Limitations

To achieve high internal validity, we included only RCTs in our exploration of the effects of ethanol lock solutions for patients with CVC. The review was conducted according to the prespecified protocol, which has been registered in PROSPERO. However, as is the case with all systematic reviews, several important limitations should be noted.

First, the reporting of the included studies themselves was incomplete. The majority of the studies failed to specify the method of randomization, use appropriate allocation concealment procedures, and ensure blinding of trial participants; these represent significant methodological limitations that may have biased the results.

Second, there was significant heterogeneity within the trials. For example, ethanol dwell times varied from 2 minutes to 48 hours. However, given the small number of eligible studies, we did not perform meaningful subgroup analysis, which is important given the heterogeneity of the study. We propose the need for further studies to explore the preventive effect and safety parameters of ethanol at different dwell times, if possible. Admittedly, with only seven small trials, it is difficult to use techniques such as funnel plots to examine the possibility of publication bias.

Moreover, in our meta-analytic review, no data were available regarding adverse reactions associated with long-term use of ethanol locks, which may occur when they are used for the prevention of CRBSI. The major reason may be the relatively short duration of follow-up of the included studies. It would be preferable to address this type of research question using long-term prospective studies.

## Conclusion

Ultimately, despite these weaknesses, our quantitative literature review of RCTs provides clear support for the efficacy of ethanol lock solutions as a promising option for the prevention of CRBSI in patients with CVC. However, more attention should be paid to the uniformity of the ethanol lock procedure and toxic effects after long-term ethanol lock exposure.

## Supporting information

S1 Text Search strategy used in PubMed. (PDF)

S2 Text PRISMA 2009 checklist. (DOC)

S1 Table Characteristic of the included studies. (XLS)

S1 Fig Effect sizes of ethanol locks on exit site infection compared to heparin. (TIF)

S2 Fig Effect sizes of ethanol locks on catheter dysfunction compared to heparin. (TIF)

S3 Fig Effect sizes of ethanol locks on removed catheters compared to heparin. (TIF)

S4 Fig Effect sizes of ethanol locks on thrombosis compared to heparin. (TIF)

S5 Fig Effect sizes of ethanol locks on mortality compared to heparin. (TIF)

S6 Fig Effect sizes of ethanol locks on CRBSI compared to 0.9% NaCl. (TIF)

S7 Fig Effect sizes of ethanol locks on mortality compared to 0.9% NaCl. (TIF)
