# Supplementary material for: Ethanol locks for the prevention of catheter-related infection in patients with central venous catheter: A systematic review and meta-analysis of randomized controlled trials
Source: PLoS One. 2019 Sep 12;14(9):e0222408. doi: 10.1371/journal.pone.0222408 (PMC6742384; doi:10.1371/journal.pone.0222408)
Supplement: S1 Text — (PDF) [file pone.0222408.s001.pdf]

PubMed:

#1 “Ethanol”[Mesh]

#2 “Alcohols”[Mesh]

#3 alcohols* [Title/Abstract]

#4 ethanol [Title/Abstract]

#5 #1 OR #2 OR #3 OR #4

#6 “Catheter-Related Infections”[Mesh]

#7 “Infections”[Mesh]

#8 “Bacteremia”[Mesh]

#9 infection* [Title/Abstract]

#10 bacteremia* [Title/Abstract]

#11 bacteraemia* [Title/Abstract]

#12 CRB [Title/Abstract]

#13 CRI [Title/Abstract]

#14 CRBSI [Title/Abstract]

#15 #6 OR #7 OR #8 OR #9 OR #10 OR #11 OR #12 OR #13 OR #14

#16 lock*[Title/Abstract]

#17 “Randomized Controlled Trial”[Publication Type]

#18 “Randomized Controlled Trial as Topic” [Mesh]

#19 random*[Title/Abstract]

#20 #17 OR #18 OR #19

#21 #5 AND #15 AND #16 AND #20
